# The political economy of euro area sovereign debt restructuring

**DOI:** 10.1007/s10602-021-09327-9

**Published:** 2021-02-10

**Authors:** Friedrich Heinemann

**Affiliations:** 1 ZEW Mannheim, L7 1, 68161 Mannheim, Germany; 2 University of Heidelberg, Heidelberg, Germany

**Keywords:** Sovereign debt restructuring mechanism, Banking regulation, EMU reform, Fiscal union, H63, H87, F53

## Abstract

The establishment of a sovereign debt restructuring mechanism (SDRM) is one of the important issues in the academic debate on a viable constitution for the European Monetary Union (EMU). Yet the topic seems to be taboo in official reform contributions to the debate. Against this backdrop, the article identifies the SDRM interests of key players, including the European Commission, the European Parliament, the European Central Bank and national governments. The empirical section takes advantage of the recently established EMU Positions Database. The findings confirm political economy expectations: Low-debt countries support an EMU constitution that includes an insolvency procedure whereas a coalition of high-debt countries and European institutions oppose it. The analysis points towards a possible political-economic equilibrium for coping with sovereign insolvencies: an institutional set-up without an SDRM and with hidden transfers. Recent European fiscal innovations in response to the Covid-19 solvency shock confirm this prediction.

## Introduction

The potential role of debt restructuring mechanisms in the constitutional set-up of the European Monetary Union (EMU) has received considerable attention from academics. Following earlier reflections on developing countries and the IMF (Krueger 2002), the euro-area debt crisis kicked-off a large and still growing literature on how to organize debt restructuring for the euro area in an orderly way (Bénassy-Quéré et al. 2018; Fuest et al. 2016; Gianviti et al. 2010; Gros and Mayer^2010^; Mody 2013).

Academia’s keen interest in sovereign debt restructuring mechanisms (SDRM) for the euro area stands in sharp contrast to the topic’s neglect among EU institutions. The European Commission’s 2017 “Reflection Paper on the Deepening of the Economic and Monetary Union” is an illustrative example (European Commission 2017). The paper is highly ambitious with respect to the completion of the banking union (European Deposit Insurance Scheme), new debt instruments (sovereign bond-backed securities), a new macroeconomic stabilization function (e.g. a European unemployment insurance scheme), the establishment of a European Monetary Fund (EMF) or the establishment of a euro area Treasury. At the same time, it does not include any hint to the possible role and organization of an SDRM. The Commission’s disregard of issues related to debt restructuring continued in the “Saint Nicolaus’ package”, the comprehensive set of detailed EMU reform plans presented by the European Commission in December 2017. Once again, it offered no solutions on how to cope with an insolvent euro area government. Even the massive fiscal solvency shock as a result of the Covid-19 pandemic has so far not triggered a new European SDRM debate.

One obvious explanation for this reticence of European political institutions is the fear that the mere existence of an SDRM could destabilize government bond markets. But this does not suffice to explain why European institutions hardly discuss the issue. Recent academic studies on a European SDRM are fully aware of its challenges and have offered various strategies for coping with the problems (Bénassy-Quéré et al. 2018; Fuest et al. 2016).

This article provides political economic explanations for the divergent positions taken by various parties on explicit sovereign debt restructuring in a future EMU. It covers the following key players: the European Commission, the European Parliament, the European Central Bank (ECB) and the governments of high-debt and low-debt euro area countries.

The political economy of an SDRM has received substantial academic attention in the context of the IMF model for developing and emerging countries proposed in 2002 (Krueger 2002). The IMF model wanted to address the procrastination problems with over-indebted economies. Too often, countries with unsustainable debt levels delayed restructuring to the detriment of both creditors and the domestic economy. Proponents of an SDRM wanted to promote a predictable, orderly and rapid restructuring that could overcome the coordination failures of ad hoc debt negotiations. After an intense debate, the Krueger SDRM model failed to gain sufficient political support from key players (Quarles 2010; Roubini and Setser 2004; Setser 2010). Borrowing developing countries were afraid to lose sovereignty as the IMF would have gained jurisdiction over domestic-law debt and exerted an even stronger impact on domestic policies. Creditor countries were concerned about the moral hazard effects of possibly too quick and generous restructurings and, not unlike borrowers, the growing IMF power. Moreover, the US administration under President George Bush favored contractual market-based solutions instead of a statutory restructuring mechanism and, therefore, pushed the use of Collective Action Clauses (CACs) in bond contracts. The shifting attention towards CACs brought the IMF-centered SDRM debate to an end (Gelpern and Gulati 2013).

CACs can alleviate restructuring negotiations as they define creditor voting rules and qualified majorities of bondholders that bind all bondholders within the same issuance to the restructuring terms. However, CACs are not at all a full substitute for a fully developed SDRM since such a mechanism goes far beyond the definition of voting rules for bondholders (Krueger and Hagan 2005; Roubini and Setser 2004): An SDRM establishes a comprehensive framework to prepare, negotiate and execute a sovereign debt restructuring. It sets up institutions and committees for the restructuring negotiations; it covers a wider range of sovereign debt instruments beyond bonds; like in private insolvency procedures, an SDRM defines debtor information requirements and debtor protection with equal-treatment of diverse creditors; it provides temporary liquidity to the creditor over the period in which the restructuring procedure is ongoing; and it sets incentives for prudent and responsible policies in the transition phase.

This older IMF debate is a starting point for this analysis, which considers the support for a European SDRM in the institutional context of the euro area. Like for the IMF SDRM, the debtor-creditor antagonism plays a key role in in the current European setting. However, other issues of the earlier debate are of less relevance in Europe today. With strong supranational EU institutions, the shift of power from the nation states to a higher level is already far advanced in Europe. Hence, one of the key counter-arguments against the IMF SDRM—a loss of national sovereignty—is much less convincing in the current European debate.

This study finds that the diverging positions are consistent with institutional self-interests. For the European Commission and the European Parliament, the absence of debt restructuring increases the need for large and permanent centralized fiscal instruments in line with the centralization interests of these institutions. The ECB position is ambivalent. From a monetary policy perspective, the ECB has a strong interest in a smooth debt restructuring whose burden falls on private investors in order to avoid any monetary involvement in a bail-out. But the ECB today is massively exposed to euro area sovereign debt and might, therefore, fear write-offs that are likely to violate the legal ban on monetary financing. Moreover, the ECB likely fears the fall-out of restructuring for banking stability given its banking supervision mandate.

For euro area governments, the case is asymmetric, but there is room for compromise. Low-debt countries fear the burden of transfers if high-debt countries become insolvent and no credible restructuring mechanism exists. Conversely, high-debt countries with a non-negligible risk of future insolvency prefer transfers over a debt cut, with all its economic and political costs. The analysis concludes that a non-transparent transfer arrangement like the one applied in Greece could be an acceptable compromise for everyone. Hidden transfers need substantive and permanent fiscal instruments, which are in the centralizing interests of the Commission, the Parliament and the European bureaucracies. Hidden transfers avoid visible problems for the ECB balance sheet and are not at odds with its banking supervision mandate. Moreover, hidden transfers are in the interests of high-debt countries because they effectively reduce the debt-service burden. Finally, non-transparency helps limit the political costs for incumbent governments in sustainable debt countries who have to bear the burden of the effective bailout.

The next section explains the role of transfers and debt restructuring in a European fiscal union with regard to two inconsistent taboos in the current reform debate. Section 3 looks in detail at the interests of important institutions. The empirical section introduces the EMU Positions Database and tests several predictions. The final section examines the implications for a possible compromise and discusses recent fiscal innovations in the corona pandemic in the light of the paper’s findings and predictions.

## Two inconsistent taboos

Talk of sovereign debt restructuring is not the only taboo in the euro reform debate. None of the important official EMU reform templates includes any explicit transfer element. Any new fiscal capacities (e.g. European unemployment insurance) or loan instruments (European Monetary Fund) are presented as part of an *insurance* narrative. The defining element of any insurance scheme is that there is no systematic ex ante redistribution (from rich to poor or from low-debt to high-debt countries). But an institutional arrangement that excludes transfers and sovereign debt restructuring simultaneously is inconsistent (Rodden 2017). A consistent design can either exclude sovereign debt restructuring or exclude a transfer solution. But it cannot coherently exclude both elements at the same time if there are (or could be in the future) cases of sovereign insolvencies. Conceptually, a country is insolvent if the present value of future revenues does not suffice to balance the current debt stock and the net present value of future expenditures, even for the maximum feasible fiscal adjustment (Das et al. 2012).

Three basic solutions are available for an insolvent country:

- *Unexpected positive solvency shocks* Examples are structural reforms that are surprisingly courageous and successful in boosting an economy’s growth potential, technological innovations, the discovery of natural resources, and improvement in terms of trade. Positive shocks of any such type could turn a situation of insolvency into solvency.
- *Debt restructuring (at the expense of private creditors)* A debt restructuring that reduces the net present value (NPV) of debt service obligations can restore debt sustainability. NPV effects can be achieved through multiple instruments (Das et al. 2012): haircuts that reduce the face value of a debt obligation; a maturity extension that postpones the repayment obligation; and interest rate reductions. All these instruments involve redistribution from the lender to the borrower.
- *Transfers (at the expense of other jurisdictions’ taxpayers)* In the absence of a positive solvency shock, the only alternative to private creditor debt restructuring are transfers from other sovereigns (Zettelmeyer et al. 2013). The insolvent country can be rescued through transfers from countries, international institutions and central banks. Transfers can have various forms and be explicit or implicit and, hence, have very different levels of salience. A cash bail-out from other countries would be a particularly salient and direct way to reduce the debt service NPV. An identical effect can be achieved through preferential loan assistance. Financial assistance includes a transfer element whenever interest rates do not include a risk spread that fully reflects the debtor’s credit risk. The transfer element in any such financial assistance amounts to the face value of the financial assistance minus the NPV of the debtor country’s repayment obligation calculated on the basis of a risk-adequate discount rate.

One important caveat relates to the difficult distinction between illiquidity and insolvency. The euro area debt crisis with it panic-driven contagion in the 2010–2012 period has demonstrated that countries can fall victim to liquidity crises that would be otherwise manageable in calm market environments. De Grauwe and Ji (2013a) point out that a liquidity crisis can turn into a solvency crisis if, for example, illiquidity forces a country to pursue austerity measures that damage a country’s long-run growth. According to the multiple equilibria theory, a self-fulfilling prophecy may emerge in which a country becomes insolvent because investors fear insolvency (De Grauwe and Ji 2012). In such a case, preferential financial assistance may prevent insolvency in the first place.

De Grauwe and Ji clarify that past or future euro area insolvencies are not necessarily the result of a self-fulfilling prophecy. In their multiple equilibria model, they show that the distinction between illiquidity and insolvency is blurry only for an intermediate range of fundamental indicators. If the fundamentals deteriorate below a critical level, even optimistic market sentiment cannot restore solvency (De Grauwe and Ji 2013b). For these cases the only remaining decision is whether to pursue private debt restructuring or transfers (of whatever type).

So far, there has been one instance in the euro area where debt sustainability was fundamentally lacking without reasonable doubt: namely, Greece in 2010–2011 (Zettelmeyer et al. 2013). The experience with Greece confirms that any such situation must trigger a debt restructuring or a transfer solution. Interestingly, Greece underwent both debt restructuring and transfers. In 2012 the Greek “private sector involvement” (PSI) restructured private Greek debt with a face value totaling more than 100 per cent of Greek GDP. Depending on discount assumptions, the NPV loss imposed on private creditors was 50 per cent or higher (Zettelmeyer et al. 2013). However, the PSI was insufficient to restore solvency so that substantial implicit transfers were required as well. These were given by means of preferential financial assistance from EU member countries (direct bilateral loans), ECB bond purchases through the Securities Market Program (SMP), the European Financial Stability Facility (EFSF), and the permanent European Stability Mechanism (ESM) (Buchheit and Gulati 2018).

Given the current state of public finances in the EMU, more cases of fundamentally insolvent euro countries are likely in the future. In its 2020 Debt Sustainability Monitor published before the outbreak of the Covid-19 pandemic, the European Commission identifies persistent fiscal sustainability risks and identifies seven EU countries “at high fiscal sustainability risk in the medium-term” including five euro area members (Belgium, Spain, France, Italy, and Portugal: European Commission 2020a). This debt sustainability analysis explicitly took account of the downward trend in government interest rates, which supports debt sustainability. Undoubtedly, with the pandemic and its massive fiscal and economic fallout, risks for future insolvencies in the euro area have further increased since high-debt countries such as Greece, Italy, Spain and France have experienced a particularly severe and probably lasting economic damage from the pandemic (European Commission 2020b).

If it is to develop a realistic overall strategy, Europe must prepare for new sovereign insolvencies beyond Greece. Rejecting the SDRM for euro area countries with the argument that insolvencies will not occur in the future is wholly unconvincing. Given Europe’s currently further deteriorating fiscal conditions, it must either open the way for debt restructuring or accept (implicit) transfers for future cases of insolvency.

In the next sections, I consider the interests of crucial players with regard to SDRs and transfers.

## Interests of crucial players

### European Commission

One of the European Commission’s main executive responsibilities is the administration of the EU budget. From a Niskanen perspective (Niskanen 1971), the Commission, as the central European bureaucracy, has an institutional interest in increasing the European budget or developing additional European fiscal instruments under its (partial) control. It should thus have a “vested interest in centralization” (Vaubel 1994: p. 235). Over the decades, there has been ample evidence that the Commission wants to strengthen fiscal power at the European level. In past negotiations regarding the EU’s Multiannual Financial Frameworks (MFF) the Commission has proposed budgets that have been larger than the budgets finally adopted. Moreover, the Commission is a long-standing advocate of new revenue types for the EU budget. In its proposal for the next MFF covering the 2021–2027 period, it has included three news types of own resources: a share from a tax on a Common Consolidated Corporate Tax Base (CCCTB); a share of revenues from the European Emission Trading System; and a “plastic tax”, a new national contribution based on the amount of non-recycled plastic waste (European Commission 2018).

#### SDRM

An SDRM for Member States would be a building block for a decentralized fiscal constitution without the necessity of significant European involvement. Debt restructuring solves the underlying problem of national overindebtedness by imposing losses on private creditors. As such it is the necessary condition to avoid open or hidden transfers from other Member States or the EU to an insolvent country. An insolvency system may still give some responsibilities to European institutions. In such a scenario, the Commission or another European institution could play a role in orchestrating the settlement. In addition, there might be the need for short-run liquidity assistance to a Member State in distress during restructuring negotiations (Fuest et al. 2016). With a credible SDRM in place, there is no need to involve the European budget over a longer time period to cope with insolvent Member States.

From the point of view of an institution whose interests lie in centralization, therefore, the establishment of a system with swift restructuring for overindebted countries will not be the preferred reform scenario. Instead, the central European bureaucracy is more likely to prefer reforms that will foster the growth of the EU budget or alternative euro area fiscal instruments. From this angle, the EU bureaucracy could see unsustainable debt in a Member State as an opportunity to establish permanent bailout instruments at the European level.

#### Transfers

In principle, transfers could also flow through horizontal payments among Member States without any EU level involvement. But horizontal transfers are not attractive for donor countries due to resistance among voters (see below). Furthermore, there is no tradition of horizontal transfers in the European integration process. All existing significant EU transfer schemes are vertical: Member States contribute to the EU budget that pays out transfers mainly through its Common Agricultural Policy (CAP) and its cohesion instruments. With these precedents, the European Commission can be optimistic that any transfer approach to an overindebted euro area country will depend greatly on the European budget and/or other fiscal capacities at the European level.

### European Parliament

The European Parliament is obviously harder to position in the reform debate given the substantial heterogeneity of its individual members. However, in all fiscal debates involving the EU budget or the need for new European revenue sources, the EP has an institutional interest in new and larger European fiscal instruments. Hence, its average position should be similar to that of the Commission, i.e. contra SDRMs and pro EU-level transfers and other fiscal instruments (provided that the Parliament has some control over them).

### European Central Bank

During past crises, the ECB has assumed more responsibilities and introduced new unconventional instruments through which it has operated on secondary markets for government bonds, giving it significant importance for financing euro area governments (Drudi et al. 2012). In 2010, the ECB established and activated the Securities Market Program (SMP), which purchased government bonds from crisis countries. At the height of the debt crisis in summer 2012, the ECB Council initiated the Outright Monetary Transactions (OMT) program, which supports countries that have an agreement with the ESM. Though the OMT has never been activated, its mere existence has played a major role in the post-2012 reduction of risk premiums in government bond markets. From 2015 until the end of 2018 and again since November 2019, the ECB and the eurozone national central banks have purchased euro area government bonds under the Public Sector Purchase Program (PSPP) (European Central Bank 2015). For the allocation of purchases across countries the ECB Council has committed to stick to the national shares in the ECB capital key. In March 2020, as a reaction to the pandemic, the ECB Council has established another substantive asset purchase program, the Pandemic Emergency Purchase Program (PEPP) that buys European sovereign bonds. Accumulated stocks of sovereign bonds in the Eurosystem balance sheets have reached 3.2 trillion euro at the end of 2020 and purchases both from the PSPP and the PEPP increasingly diverge from the ECB capital key with a significant overweight of the high-debt countries’ securities (Havlik and Heinemann 2020a).

The second major extension of ECB responsibilities is the new role within the European Banking Union, giving it direct supervisory responsibility for large systematically important banks (Howarth and Quaglia 2014).

In terms of the ECB’s institutional interests, the extension of responsibilities is a mixed blessing. The new role in banking supervision implies higher budgets and an increase in the number of staff which, from a Niskanen perspective, is a welcome development for career opportunities and bureaucratic morale (Vaubel 1997). On the other hand, preference formation on euro area developments becomes more complex in view of possible trade-offs between monetary policy objectives and financial stability. As a monetary policy institution, the ECB is responsible for keeping inflation close to its two-percent objective. As a supervisory institution, the ECB is responsible for banking stability. Major new banking crises would raise questions about the effectiveness of ECB’s supervisory branch.

#### SDRM

The ECB’s monetary and supervisory functions have an impact on how it evaluates the prospect of an SDRM. Both in the euro area debt crisis and the Covid-19 pandemic, the ECB has emerged as the de facto lender of last resort to governments threatened by a loss of market access. Financial assistance to a country in a mere liquidity crisis is temporary and does not impose major risks on money supply and inflation. The case is different if the ECB buys the bonds of an insolvent country. This does not only bring the risk of substantial future write-offs. A systemic crisis could force the central bank to continue purchases even if it impairs the bank’s ability to control the money supply. To prevent this “fiscal dominance” scenario, the ECB’s monetary policy division should have a genuine interest in the existence of a workable insolvency procedure for euro area governments. From this perspective, an SDRM would be the alternative to a bailout with ECB resources.

The interests of the ECB’s supervisory division are different. Euro area banks are heavily exposed to government bonds and biased towards their respective countries. Exposure is even larger for banking systems in countries with high government debt. Evidence from the debt crisis shows that public banks and banks that are politically connected tend to buy more government bonds when their country is in financial stress (De Marco and Macchiavelli 2016; Ongena et al. 2016). Due to the growing nexus between governments and banks, a sovereign debt restructuring is likely to trigger new banking crises under current euro area conditions. Any crisis would challenge the ECB’s supervisory role and lead to accusations that the ECB should have addressed the sovereign-bank nexus earlier. The precedent for the link between debt restructuring and bank stability is the October 2010 “Deauville statement”, in which President Sarkozy and Chancellor Merkel called for private-sector participation. Deauville led to widening bond spreads and an aggravation of the banking crisis (Zettelmeyer et al. 2013). This suggests that the ECB’s supervisory branch would strongly oppose any sovereign debt restructuring mechanism.

An even more immediate reason why the ECB would oppose an SDRM is its large exposure to government bonds under PSPP and PEPP. The ECB has explicitly accepted a “pari passu” treatment of the Eurosystem’s holdings, i.e. the ECB and national central banks are treated in the same way as private investors (European Central Bank 2015, p. 2). If any euro country is subjected to an SDRM, the Eurosystem suffers the same losses as private investors. There is a debate about the extent to which central banks should actually care about losses and whether a central bank’s capital is at all relevant for monetary policy (Diessner 2018). But it is obvious that losses would be a highly political issue, significantly damaging the reputation of the ECB as an institution and the persons in charge. Explicit losses from write-offs would be highly salient. Hence, with significant PSPP/PEPP stocks on the Eurosystem’s balance sheets for many years to come, the ECB’s views on SDRM are likely to be heavily influenced by concerns about how losses will affect it.

In sum, the ECB’s supervisory role and the Eurosystem’s large exposure to government bonds should make it oppose an SDRM. Yet its monetary policy function also incentivizes it to welcome such a system because it eliminates the threat of fiscal dominance.

#### Transfers

Because the ECB is likely to take a skeptical position on SDRM, we can assume that it will advocate a fiscal transfer approach. Like SDRM, fiscal transfers are an alternative to a monetary bailout and shield the central bank against the threat of fiscal dominance. For the ECB, it is a much more attractive alternative with less risky side effects. Fiscal transfers that solve the problem of an insolvent euro country address all the ECB’s concerns. Transfers avoid losses for government bond holders and protect the ECB from losses on its PSPP holdings. Fiscal transfers that restore government debt sustainability also protect the banks that are heavily exposed to their home country’s debt. This is a particularly attractive consequence in view of the ECB’s supervisory function.

### Governments of high-debt EMU countries

One obvious determinant of national government preferences is the public debt level. Countries with unsustainable debt levels have different perspectives from that of fiscally healthier countries. High public debt and the pressure to consolidate put political systems under stress. Incumbent governments have to confront voters with unpleasant choices about which groups should bear the burden of adjustment. The essential problem of deteriorating market access and the threat of insolvency is that the strategy of shifting the burden towards future generations becomes increasingly expensive or even impossible.

Hence, governments of high-debt countries have an interest in European institutions that alleviate fiscal constraints and help reduce the need for adjustments and the resulting political costs. Both debt restructurings and transfers can give countries fiscal leeway. However, there are good reasons to think that transfers from third parties or supranationals are less politically costly than restructuring.

#### SDRM

Debt restructuring shifts the burden of adjustment from citizens to creditors. From the perspective of taxpayers, public employees and transfer recipients, restructuring is preferable to more austerity. To national policy-makers, the political benefit from restructuring is that it protects politically influential voter groups against more fiscal consolidation. Nevertheless, a default still entails significant domestic political costs.

First, households and banks in the euro area are among the most important creditor groups of domestic government debt. From restructuring, citizens may gain as taxpayers, but they could lose as savers. Unlike developing countries that receive most of their financing from external creditors, euro area countries place a substantial share of government bonds domestically. With haircuts or other restructuring measures, therefore, substantial losses of wealth can arise for domestic groups. A particular feature of euro area countries is a strong home bias in government bond markets combined with a pronounced bank-government nexus. As a result, domestic banks are heavily exposed to their own government. The exposure is particularly large in highly indebted euro countries, especially after the debt crisis (De Marco and Macchiavelli, 2016; Ongena et al. 2016). Given this bank-sovereign-nexus, the losses incurred by domestic savers depend on the effectiveness and credibility of domestic deposit insurance. The establishment of a European Deposit Insurance Scheme (EDIS) with the full European collectivization of risk would make an important difference. As long as EDIS does not exist, however, debt restructuring poses a substantial risk for domestic savers in the event of government default.

Second, experience shows that government defaults create significant “domestic collateral damage” (Panizza et al. 2009) beyond the immediate losses for creditors. Empirical studies (as surveyed in Panizza et al. 2009) have found that substantial output losses accompany a default. There are two channels to explain these real losses: The default reveals bad news about an economy and a country, or it leads to capital flight and lowers market sentiment.^1^

On account of the domestic costs, sovereign debt restructuring is not particularly attractive to politicians and senior civil servants. Sovereign insolvency sends a very strong negative signal to voters about the competency of the incumbent government. This reasoning is supported by empirical evidence. Borensztein and Panizza (2009) show that defaults are typically followed by large decreases in electoral support for incumbent parties, and subsequent changes to leadership are much more common than under normal circumstances.

National politicians’ opposition to debt restructuring begins long before the debt restructuring actually occurs. An introduction of an orderly SDRM will induce government bond pricing that is more sensitive to changes in the fiscal and economic fundamentals. Countries with a risky fiscal outlook will pay an immediate price for any euro area reform that makes debt restructuring more likely. Hence, the mere existence of a restructuring mechanism can impose constraints on high-debt countries even when an actual default is still nothing more than a distant possibility. This is why the governments of high-debt countries are likely to oppose an SDRM even if their countries have not yet reached a state of insolvency.

#### Transfers

For high-debt countries, wealth transfers are obviously more attractive than the prospect of debt restructuring with all its political costs. If large enough, transfers can restore debt sustainability and, unlike debt restructuring, avoid wealth losses for domestic creditors because they shift the burden to taxpayers in other countries. Moreover, transfers avoid the risk of banking crises. The transfer of wealth from abroad has an income effect for the domestic population and improves the country’s creditworthiness. This is likely to stabilize economic growth.

However, transfers often come in combination with conditionality. Much of the preferential financial assistance in the European debt crisis has diminished the autonomy of EU nation states. Countries that wanted to benefit from ESM assistance and, possibly, subsequent ECB assistance through the OMT program had to sign detailed memoranda of understanding on consolidation measures and structural reforms defined by the “Troika” (European Commission, ECB, and IMF). This conditionality is politically costly, as voters tend to hold the incumbent government responsible for accepting and implementing the conditionality that perceived as intrusive.

The most favored solution among high-debt governments is, therefore, the unconditional transfer. Politicians in high-debt countries will favor any reform idea that attenuates the conditionality of existing aid instruments or creates new instruments that imply unconditional transfer elements. As with SDRMs, transfers have an immediate advantage long before the money flow starts. The prospect of transfers immediately improves a high-debt country’s creditworthiness. This means lower risk spreads on government bonds and a looser budget constraint that creates immediate opportunities for redistributing future resources to current taxpayers, public employees and benefit recipients.

### Governments of low-debt EMU countries

In general, fiscally sound countries see transfers and debt restructuring asymmetrically to high-debt countries. The economic burden of transfers falls on solvent countries’ citizens. Of course, transfers are a regular feature within nation states with regional imbalances. But even then, transfers are often contentious and an important driver for secessionist movements in donor regions (Gehring and Schneider, 2020). In Europe, systematic transfers are likely to have even higher political costs for donor jurisdictions because the sense of solidarity is normally stronger within countries than between countries. For example, the subject of the national “net balances” vis-à-vis the EU budget is one of the most notorious conflicts between Member States and regularly dominates EU budget negotiations and the Multiannual Financial Frameworks (MFF) (Asatryan et al. 2020; Benedetto et al. 2020).

We can therefore assume that fiscally sound countries will strongly resist any strategy that passes on the costs of other Member States’ debt to their own voters. Instead, these countries will tend to prefer austerity in high-debt countries to allow for repayment as contracted (Copelovitch et al. 2016). But this assumption requires several qualifications.

#### SDRM

A sovereign debt restructuring obviates the need for a bailout and hence is in the interest of countries whose citizens would otherwise have to pay for a bailout. Of course, some costs of restructuring may spill over to other countries as well. The spillover is direct if domestic banks are exposed to debts of the defaulting country. For example, on the eve of the Greek debt crisis in 2009, French and German banks had the largest exposure to Greek sovereign bonds among euro countries (Guiso et al. 2016). The spillover is indirect if there is “collateral damage” from restructuring with negative financial or economic cross-border effects. So far, the EMU debt crisis with the only restructuring precedent of the Greek PSI has not led to high collateral damage for low-debt countries. On the contrary, the capital flight from crisis into safe haven countries has arguably increased the latter’s growth and relaxed their budgetary constraints thanks to falling interest rates. It is uncertain whether cross-border damage will be larger in the wake of future EMU defaults, especially if countries larger than Greece default. However, the lesson of the last decade is that fiscally sound countries do not pay a significant price for defaults elsewhere in the monetary union. To further minimize the risk, low-debt countries are likely to push for the establishment of well-prepared and orderly restructuring processes that further minimize the risk of cross-border collateral damage. Moreover, low-debt countries also have an interest in an SDRM long before any restructuring takes place, because the availability of this mechanism will benefit them by fostering stronger interest rate discrimination between fiscally risky countries and fiscally sound countries. This is to the immediate benefit of fiscally healthy countries.

#### Transfers

Transfers are plainly unpopular among countries that stand to be net payers. However, the political costs of transfers depend on the transparency of the transfer instrument. The most transparent type would be a horizontal cash transfer from a low-debt to a high-debt country with immediate impact on current budgets. Less visible transfers include guarantees that enable an institution like the ESM to raise funding and channel it to high-debt countries at preferential interest rates for long maturities. In the event that transfers are not avoidable, low-debt countries’ governments have a clear interest in using non-transparent types in order to minimize voter disproval.

## Evidence

### Anecdotal evidence

There is ample anecdotal evidence that the Commission behaves in line with aforementioned predictions. The Commission’s “Reflection Paper on the Deepening of the Economic and Monetary Union” from May 2017 develops several reform scenarios for the future of the EMU (European Commission 2017). It provides a wealth of ideas for new fiscal instruments but is completely silent on the role of sovereign debt restructuring. The reflection paper proposes several new fiscal instruments: a fiscal backstop to the Single Resolution Fund, possibly through a credit line from the ESM; sovereign bond-backed securities (“safe assets”), possibly combined with favorable regulatory incentives; a new “macroeconomic stabilization function”; the establishment of a “European Treasury”; and the set-up of a European Monetary Fund (EMF). The reflection paper pays attention to risk reduction in bank balance sheets with a focus on non-performing loans. It mentions the problem of large domestic government exposures among national banking systems, yet it takes a defensive position on the risk-free status for sovereign bonds in bank capital requirements. The regulatory privilege for public borrowers is an obstacle for the introduction of SDRMs and would have to be phased out to prepare a viable insolvency procedure (Fuest et al. 2016). By contrast, in the eyes of the Commission, the risk-free status of sovereign bonds “is justified by their particular role in funding public expenditure and in providing a low-risk asset for the financial system of the country concerned” (European Commission 2017: p. 22).

The Commission’s aversion to any SDRM is also visible in its reflections on a possible future EMF. From the beginning, the academic debate on the establishment of the EMF has been closely connected to the idea of an orderly procedure for sovereign debt restructuring (Gros and Mayer 2010; Rodden 2017; Weder di Mauro and Zettelmeyer 2017; Wyplosz 2017). In the Commission’s view, the EMF has no role to play in that respect. In its proposed draft regulation from December 2017, the EMF does not superintend or moderate a debt-restructuring mechanism; nor does it have any role in the debt sustainability analysis (DSA) that precedes EMF loans. Rather, the DSA is performed by the Commission and the ECB. A comparison of the Commission’s draft EMF regulation with the existing ESM Treaty (to be replaced by the new regulation) reveals that the Commission even intended to eliminate an existing debt restructuring rule. The Commission proposal dropped the requirement for the inclusion of CACs in all euro area government bonds as they currently exist under the ESM Treaty (Gasparotti et al. 2018). Hence, the Commission did not only resist a comprehensive statutory restructuring mechanism in the EMF regulation; it also revealed the intention to eliminate even milder instruments such as CACs that facilitate a sovereign debt restructuring.

Thus, the Commission’s position on EMU reform is consistent with the prediction that the institution’s centralizing interests lead it to prefer a European fiscal union without credible instruments for sovereign debt restructuring. Instead, the Commission’s implicit answer to overindebtedness is financial assistance from various new European fiscal instruments.

A similar finding exists for the ECB. The ECB staunchly opposes any attempt to establish an orderly SDRM for the euro area. So great is its resistance that the last ECB president, Mario Draghi, regarded it as a taboo issue. Asked in May 2017 about the consequences of a euro area Member State in need of debt restructuring, the ECB president answered: “We don’t want to speculate on the probability of things that have no chance of happening. Why are you asking me that?” (cited after: Buchheit and Gulati 2018, p. 65). To date, the ECB has never issued a public statement on the matter. By contrast, ECB representatives have regularly called for the establishment of new fiscal instruments to stabilize the euro area—another indication that the ECB strongly prefers transfers to debt restructuring for future sovereign insolvencies.

There is evidence that low-debt countries support an SDRM. For instance, the German and French governments have taken a position on the ESM and EMF that is different to that of the Commission. In their joint “Meseberg Declaration” from June 2018, both stressed the importance of debt sustainability analysis in any liquidity support decision, proposed improving CACs instead of deleting the requirement, and recommended the ESM “to facilitate the dialogue between its Members and private investors, following IMF practice” (Press and Information Office 2018). Essentially, this would amount to assigning the ESM a moderator role in debt restructuring negotiations. Moreover, in a joint letter, the finance ministers of the “Hanseatic League” countries—the EU Member States from Scandinavia, the Baltic States, the Netherlands, and Ireland—explicitly called for an EMF responsibility for an SDRM: “Moreover, the modalities of a strengthened framework for orderly sovereign debt restructuring in case of unsustainable debt levels should be explored as part of the set-up of an EMF” (Finance Ministers 2018).

Typically, high-debt countries take the exact opposite standpoint. In the talks to reform the ESM Treaty, the Italian government opposed any change that could facilitate the restructuring of government debt or that would end the regulatory treatment of banks’ government exposure and the zero weighting in capital requirements. At the same time, Italy demanded new central fiscal instruments such as a common European unemployment insurance mechanism and a euro area budget (Fonte and Jones 2019).

The negotiations on new fiscal instruments designed to stabilize EU countries during the corona recession provide further rich support for various predictions. The highly indebted Southern European countries were highly critical on making the ESM the central vehicle for crisis support as they rejected its conditionality (Tesche 2020). The degree of conditionality was also a key dispute in the negotiations on the European reconstruction package “EU Next Generation” that will mobilize 750 billion euros in the coming years. In these negotiations, the fiscally sounder countries (“the frugal four”), led by the Netherlands, demanded strict conditionality and were keen to limit the Corona fund’s (visible) transfer elements (Schmidt 2020).

### Empirical evidence

This section empirically tests the predictions by providing classified comparisons based on the EMU Position Database of Wasserfallen et al. (2019). The database places all EU countries and European institutions (including the Commission, the Parliament, and the European Central Bank) in a policy space covering several of the disputed EMU reforms. The disputed issues include assistance to Greece, EFSF, ESM, Six-Pack, Two-Pack, Fiscal Compact, Banking Union, FTT, eurobonds, and the Five Presidents’ Report. The EMU position project team considered more than 5000 documents, from Euractiv and other quality news media to official EU and national documents and academic publications. Each positioning decision was cross-checked by a second review team. Tables 1, 2, 3, 4, 5, 6 classify EMU member countries and European institutions based on their support (small, medium, and large) for a given position and compares the average debt-to-GDP level for each of the country groups.

**Table 1:** Support for supranational arrangements such as fiscal capacity or eurobills: member states/European institutions

Small [0–33.3]	Medium [33.3–66.6]	Large [66.6–100]
LTU, MLT, FIN, NLD, IRL, AUT	DEU, FRA,	EST, LUX, LVA, SVK, SVN, ESP, BEL, CYP, PRT, ITA EP
Average debt-to-GDP level of Member States in % (2015)		
65.2	83.2	77.8

Policy space: 0: Status quo. 50: Reforms within the current treaties with possible intergovernmental arrangements outside of current treaties. 100: Moving towards fiscal federalism through supranational arrangements (e.g., fiscal capacity, eurobills)

**Table 2:** Support for single resolution fund and mutualization: member states/European institutions

Against [0–33.3]	Neutral [33.3–66.6]	For [66.6–100]
DEU, FIN, NLD, AUT	–	EST, IRL, LTU, LVA, SVK, SVN, FRA, ITA, BEL, GRC, PRT, ESP, CYP, LUX COM, ECB, EP
Average debt-to-GDP level of Member States in % (2015)		
71.0		83.5

Policy space: 0: Networks of national resolution funds without mutualization in foreseeable future. 80: Centralized Single Resolution Fund created by gradual mutualization of national resolution funds over the period of 8–10 years (covering their build-up from bank levies). 100: Centralized Single Resolution Fund created by mutualization of national funds within 8 years

**Table 3:** Support for eurobonds: member states/European institutions

Against [0–33.3]	Neutral [33.3–66.6]	For [66.6–100]
AUT, DEU, EST, FIN, LTU, NLD	LVA, MLT, SVK	BEL, ESP, FRA, GRC, IRL, ITA, LUX, PRT COM, EP
Average debt-to-GDP level of Member States in % (2015)		
56.1	49.2	102.7

Policy space: 0: No, not even in the long-term. 50: Not now, without any indication when. 100: Yes, in principle now, but only under certain conditions

**Table 4:** Support for withholding EU funds to deficit countries: Member States / European institutions

Against [0–33.3]	Neutral [33.3–66.6]	For [66.6–100]
BEL, ESP, FRA, GRC, IRL, ITA, LTU, MLT, PRT, SVN COM, EP	CYP	SVK, AUT, DEU, EST, FIN, LUX, LVA, NLD, ECB
Average debt-to-GDP level of Member States in % (2015)		
99.8	108	50.6

Policy space: 0: Opposes the withholding of EU funds. 100: Supports the withholding of EU funds when a Member State breaches deficit limits

**Table 5:** Support for IMF involvement in first Greek program: member states/European institutions

Against [0–33.3]	Neutral [33.3–66.6]	In favor [66.6–100]
FRA, ESP, ITA, GRC ECB, EP	EST, LUX, LTU, SVK, MLT, IRL, SVN, CYP, PRT, DEU, AUT COM	BEL, LVA, FIN, NLD
Average debt-to-GDP level of Member States in % (2015)		
125.6	67.0	67.9

Policy space: 0: Against IMF involvement; for EU-only rescue program. 50: Support only after Germany shifted its position in favor of IMF participation. 100: Support for IMF participation (even before Germany switched its position)

**Table 6:** Support for Greek private sector involvement: member states/European institutions

Against [0–33.3]	Neutral [33.3–66.6]	For [66.6–100]
BEL, LUX, MLT, IRL, FRA, ESP, CYP, PRT, ITA, GRC COM, ECB, EP		AUT, EST, SVK, FIN, NLD, DEU, SVN
Average debt-to-GDP level of Member States in % (2015)		
100.3		61.2

Policy space: 0: Against any private sector involvement in debt restructuring that is not purely voluntary. 20: In accordance with IMF practice, mandatory PSI only in exceptional cases, and moreover only in an “adequate" and “proportionate" form. 100: Comprehensive, considerable and mandatory involvement of the private sector in debt restructuring

Tables 1, 2, 3 contain positions on new fiscal instruments that could serve a transfer purpose: fiscal capacities/eurobills (Table 1); mutualization of the Single Resolution Fund (Table 2); and eurobonds (Table 3).

Tables 4 and 5 describe conditionality with regard to the withholding of EU funds for countries with high deficits (Table 4) and to the inclusion of the IMF in the Greek program as a credibility anchor for stricter conditionality (Table 5).

Table 6 contains positions on Greek debt restructuring via PSI (the first effort to restructure sovereign debt in the euro area and thus an important precedent for the establishment of an SDRM).

The results are strikingly in line with the predictions. For all three financing instruments (Tables 1, 2, 3) the debt levels of SDRM supporters are on average higher than the debt levels of SDRM opponents. In all cases where the position of the Commission, the European Parliament, or the ECB is identifiable it sides with the supporters and high-debt countries. This is most obvious in the case of eurobonds (Table 3), where the average government debt-to-GDP level is almost twice as high for supporters as for opponents.

The assessment of conditionality—linking EU funds with high deficits (Table 4) or the involvement of the International Monetary Fund (Table 5)—leads to the same polarization between high-debt countries (against conditionality) and low-debt countries (for conditionality). Neither the Commission nor the Parliament supports either type of conditionality. Only the ECB supports conditionality for EU transfers.

When it comes to support for PSI, empirical results also align with the predictions: High-debt countries follow all three European institutions in their opposition to Greek debt restructuring, while low-debt governments support it.

## Hidden transfers as political-economic equilibrium

Both the anecdotal and the empirical evidence confirm the expected conflict of interest between low-debt EMU countries (in favor of SDRM and opposed to transfers) on the one hand and high-debt countries, the Commission, the Parliament and the ECB (opposed to SDRM and in favor of transfers) on the other. The question is which compromise is feasible. As noted in 3.5, fiscally sound countries are more willing to consider transfers if they are non-transparent and escape voter notice in the donor countries. The model case for hidden transfers to an insolvent country is Greece, where the ESM has lengthened loan maturities and decreased interest rates. Maturity profiles are now back-loaded into the very distant future (with maximum maturities extending into the 2060s). Hidden transfers can be quantified by comparing the nominal loan with the present value of agreed interest and maturity payments using a risk-adequate discount rate. In Greece, simple model calculations point to effective transfers amounting to more than 50% of nominal loans (Buchheit and Gulati 2018).

Hidden transfers satisfy all political and economic constraints. In the case of Greece, the government and citizenry have benefitted from a sharply reduced debt service for a politically relevant time horizon. The Greek settlement sent a signal to sovereign bond markets that EMU member countries can rely on long-run financial assistance. The signal has alleviated market pressure on other high-debt euro countries whose debt sustainability is disputed. The European Commission and the Parliament have also benefitted from a new and lasting European fiscal institution (ESM), which they hope to control to some degree by passing into EU law. Moreover, the ECB is satisfied because Greece no longer endangers the stability of European banks or constrains monetary policy decisions. Finally, given the absence of an immediate budgetary impact and the scant attention it has received by the media and the general public, the ESM has reduced the level of voter anger in low-debt countries such as Germany, Netherland, and Finland. Overall, the Greek solution offers a model case for future sovereign insolvencies in the EMU.

## Conclusions

Realistically, additional cases of insolvent EMU countries cannot be excluded for the coming years in view of the poor state of public finances in numerous euro countries already before the pandemic, the unwillingness to create fiscal buffers during times of prosperity (European Fiscal Board 2020), and the massive new solvency shock that has occurred since 2020 (European Commission 2020b). If the EU rules out an SDRM, transfers are the only remaining alternative for these cases. If open transfers fail to receive political support in donor countries, hidden transfers will be the compromise that satisfies all the political and economic constraints.

Various features of the new pandemic-induced European monetary and fiscal instruments actually point into the direction of hidden transfers. With the new asset purchases under PEPP, the ECB has abandoned several earlier precautions against excessive exposure to high-debt euro countries (Havlik and Heinemann 2020b). The ECB Council has lowered the credit quality standards for eligible securities and now accepts Greek sovereign bonds that previously, due to the country’s unfavorable credit rating, were excluded. It has furthermore given up a strict allocation of purchases across countries according to the country shares in the ECB capital key and effectively overweights high-debt countries. Moreover, the ECB had to accept that the Eurosystem’s holdings of euro area government bonds surpass the blocking minority thresholds defined in the CACs. This implies that the ECB Council will have a veto power in future bondholder votes, which makes a CAC-based debt restructuring highly unlikely.

Also the financing of the EU corona recovery plan can be interpreted as a move towards a transfer solution. From the 750 billion euro package, 390 billion euros are paid out as non-refundable grants and 360 billion euros as loans. This 750 billion euro package is fully debt-financed through the issuance of EU bonds guaranteed by the EU budget. However, the EU capacity to repay the debt is ultimately secured through increased EU claims to Member State contributions (Heinemann 2020). The duration of this financial operation is very long with the repayment of maturing bonds dragging on until the year 2058. According to the binding international treaty on the EU own resource system, a long-lasting joint liability for the corona debt was agreed (Council of the European Union 2020): Whenever in the coming four decades one Member State defaults on its European financial obligation or it leaves the EU without a financial deal, its share will be distributed across the remaining solvent EU countries. Hence, the refinancing scheme for the corona debt previews additional transfers from other countries as a solution whenever a country is unable to pay. All these liability and transfer implications have almost fully escaped the public perception due to the complexity of the institutional design. It might be too early to finally judge, whether these decisions only reflect the exceptional circumstances of the pandemic or whether they signal a permanent course. However, decisions taken in the pandemic crisis are precedents for new crises. These precedents are fully in line with the expectation that hidden transfers are the most plausible European answer to future insolvencies of euro area Member States.

## Footnotes

1 Other potential sources of costs include exclusion from capital markets or higher interest rates due to a loss in debtor reputation. But these costs are empirically small because countries regain capital market access relatively quickly after debt restructuring without incurring large penalties (Panizza et al. 2009). Nevertheless, myopic politicians who only care about their immediate reelection prospects will focus on the short-run negative consequences of restructuring.

## References

[CR49] Asatryan, Z., Havlik, A., Heinemann, F., Nover, J., & Pilati, M. (2020). The net operating balances: Variants, emerging numbers and history. *European Parliament, Briefing, Requested by the BUDG Committee, PE 648.183*.

[CR1] Bénassy-Quéré, A., Brunnermeier, M., Enderlein, H., Farhi, E., Fuest, C., Gourinchas, P.-O., et al. (2018). Reconciling risk sharing with market discipline: A constructive approach to Euro area reform. *CEPR Policy Insight*, 91.

[CR5] Benedetto G, Heinemann F, Zuleeg F (2020). Strategies to Overcome the ‘Juste Retour’ Perspective on the EU Budget. European Parliament, Briefing, Requested by the BUDG Committee, PE.

[CR6] Borensztein E, Panizza U (2009). The costs of sovereign default. IMF Staff Papers.

[CR7] Buchheit LC, Gulati GM (2018). Sovereign debt restructuring in Europe. Global Policy.

[CR100] Copelovitch, M., Frieden, J., & Walter, S. (2016). The political economy of the Euro crisis. *Comparative Political Studies, 49*(7), 811–840.

[CR11] Council of the European Union. (2020). Proposal for a council decision on the system of own resources of the European union, Brussels, 10025/20, 29 July 2020.

[CR13] Das, U. S., Papaioannou, M. G., & Trebesch, C. (2012). Sovereign debt restructuring 1950–2010: Literature survey, data, and stylized facts. *IMF Working Paper 12/203*.

[CR35] De Paul G, Yuemei J (2012). Mispricing of sovereign risk and macroeconomic stability in the eurozone. Journal of Common Market Studies.

[CR37] De Paul G, Yuemei J (2013). From panic-driven austerity to symmetric macroeconomic policies in the eurozone. Journal of Common Market Studies.

[CR36] De Paul G, Yuemei J (2013). Self-Fulfilling Crises in the Eurozone: An Empirical Test. Journal of International Money and Finance.

[CR19] De Marco, F., & Macchiavelli, M. (2016). The political origin of home bias: The case of Europe. *FEDS-Working Paper No. 2016–060*.

[CR42] Diessner, S. (2018). The political economy of (European) central bank capital. *Paper Presented at the Council for European Studies Conference March 2018*.

[CR51] di Mauro, B. W., & Zettelmeyer, J. (2017). The new global financial safety net, struggling for coherent governance in a multipolar system. *Centre for International Governance Innovation, Essays on International Finance*, 4(January).

[CR14] Drudi F, Durré A, Mongelli FP (2012). The interplay of economic reforms and monetary policy: The case of the Eurozone. Journal of Common Market Studies.

[CR15] European Central Bank. (2015). Decision (EU) 2015/774 of the European central bank of 4 March 2015 on a secondary markets public sector asset purchase programme (ECB/2015/10).

[CR16] European Commission. (2017). Reflection paper on the deepening of the economic and monetary union, COM(2017) 291 of 31 May 2017, Brussels.

[CR17] European Commission. (2018). Proposal for a council decision on the system of own resources of the European union, COM(2018) 325 Final, 2.5.2018, Brussels.

[CR9] Commission E (2020). Debt Sustainability Monitor 2019.

[CR10] Commission E (2020). European Economic Forecast, Autumn 2020.

[CR18] European Fiscal Board. (2020). Annual report, Brussels.

[CR20] Finance Ministers. (2018). Letter from finance ministers from Denmark, Estonia, Finland, Ireland, Latvia, Lithuania, the Netherlands and Sweden Underline their shared views and values in the discussion on the architecture of the EMU, March.

[CR23] Fuest C, Heinemann F, Schröder C (2016). A Viable insolvency procedure for sovereigns in the Euro area. Journal of Common Market Studies.

[CR24] Gasparotti A, Minkina MA, Alice Z (2018). The ESM and the proposed EMF: A tabular comparison.

[CR25] Gehring K, Schneider SA (2020). Regional resources and democratic secessionism. Journal of Public Economics.

[CR26] Gelpern A, Gulati M (2013). The wonder-clause. Journal of Comparative Economics.

[CR21] Gianviti, F., Krueger, A. O., Pisani-Ferry, J., Sapir, A., & von Hagen, J. (2010). *A European mechanism for sovereign debt restructuring: A proposal*. Brussels: Bruegel.

[CR27] Giuseppe, F., & Gavin, J. (2019). Italy PM defends reform of euro zone bailout fund but seeks concessions. Reuters business news, 2 December 2019.

[CR12] Gros, D., & Mayer, T. (2010). How to deal with sovereign default in Europe: Create the European monetary fund now! *CEPS Policy Brief, No. 202/February 2010, Updated 17 May 2010*.

[CR28] Guiso L, Herrera H, Morelli M (2016). Cultural differences and institutional integration. Journal of International Economics.

[CR29] Havlik A (2020). and Heinemann, Friedrich (2020a), Magnitudes and Capital Key Divergence of the Eurosystem's PSPP/PEPP Purchases - Update December 2020. ZEW Expert Brief.

[CR3] Havlik, A., & Heinemann, F. (2020b). Sliding down the slippery slope? Trends in the rules and country allocations of the Eurosystem’s PSPP and PEPP. *Econpol Policy Report 21*.

[CR22] Heinemann, F. (2020). Die Überdeckung der next generation EU-Schulden im Entwurf des neuen EU-Eigenmittelbeschlusses: Ausmaß und Haftungskonsequenzen, Stellungnahme anlässlich der Anhörung des Ausschusses für die Angelegenheiten der Europäischen Union des Deutschen Bundestages am 26.10.2020, Deutscher Bundestag, Ausschussdrucksache *19*(21), 112.

[CR30] Howarth D, Quaglia L (2014). The steep road to European banking union: Constructing the single resolution mechanism. Journal of Common Market Studies.

[CR32] Krueger AO (2002). A new approach to sovereign debt restructuring.

[CR2] Anne OK, Sean H (2005). Sovereign workouts: An Imf perspective. Chicago Journal of International Law.

[CR4] Mody, A. (2013). Sovereign debt and its restructuring framwork in the euro area. *Bruegel Working Paper 2013/05*.

[CR33] Niskanen WA (1971). Bureaucracy and representative government.

[CR44] Ongena, S., Popov, A., & Van Horen, N. (2016). The invisible hand of the government: “Moral suasion” during the European sovereign debt crisis. *ECB Working Paper No. 1937*.

[CR34] Panizza U, Sturzenegger F, Zettelmeyer J (2009). The economics and law of sovereign debt and default. Journal of Economic Literature.

[CR38] Press and Information Office. (2018). Meseberg declaration, renewing Europe’s promises of security and prosperity.

[CR39] Quarles R (2010). Herding cats: collective-action clauses in sovereign debt—The genesis of the project to change Market Practice in 2001 through 2003. Law and Contemporary Problems.

[CR31] Rodden, J. (2017). An evolutionary path for a European monetary fund? A comparative perspective. *European Parliament In-Depth Analysis, May*.

[CR40] Roubini N, Setser B (2004). The reform of the sovereign debt restructuring process: Problems, proposed solutions, and the Argentine episode. Journal of Restructuring Finance.

[CR41] Schmidt VA (2020). Theorizing institutional change and governance in European responses to the covid-19 pandemic. Journal of European Integration.

[CR43] Setser, B. (2010). The political economy of the SDRM. In B. Herman, J. A. Ocampo, & S. Spiegel (Eds.), *Overcoming Developing Country Debt Crises* (pp. 317–346). Oxford: Oxford University Press.

[CR45] Tesche, T. (2020). The European union’s response to the coronavirus emergency: An early assessment. *LSE Europe in Question Discussion Paper Series 157*(157).

[CR46] Vaubel R (1994). The public choice analysis of european integration: A survey. European Journal of Political Economy.

[CR47] Vaubel R (1997). The bureaucratic and partisan behavior of independent central banks: German and international evidence. European Journal of Political Economy.

[CR48] Wasserfallen F, Leuffen D, Kudrna Z, Degner H (2019). Analysing European union decision-making during the eurozone crisis with new data. European Union Politics.

[CR8] Wyplosz, C. (2017). A European monetary fund? *European Parliament In-Depth Analysis, May*.

[CR50] Zettelmeyer J, Trebesch C, Gulati M (2013). The Greek debt restructuring: An autopsy. Economic Policy.

